# Assessment of Quality of Life in Patients with Chronic Oral Mucosal Diseases Using the Indonesian Version of the Chronic Oral Mucosal Disease Questionnaire-15 (COMDQ-15)

**DOI:** 10.3390/dj12080258

**Published:** 2024-08-14

**Authors:** Febrina Rahmayanti, Ratna K. Indrastiti, Yuniardini S. Wimardhani, Sherlyana Jozerizal, Dovian Emely Suteja, Rani Handayani, Paswach Wiriyakijja

**Affiliations:** 1 Department of Oral Medicine, Faculty of Dentistry, Universitas Indonesia, Jakarta 10430, Indonesia; ratna.kumala02@ui.ac.id (R.K.I.); yuniardini@ui.ac.id (Y.S.W.);; 2 Oral Medicine Specialist, Tangerang City Regional Public Hospital, Tangerang City 15117, Indonesia; 3 Department of Oral Medicine, Faculty of Dentistry, Chulalongkorn University, Bangkok 10330, Thailand

**Keywords:** quality of life, chronic oral mucosal diseases, COMDQ-15

## Abstract

Introduction: Chronic oral mucosal diseases (COMDs) can significantly impair the quality of life (QoL) of affected individuals. Monitoring the overall disease’s impact and the efficacy of treatments requires the use of the Chronic Oral Mucosal Diseases Questionnaire-15 (COMDQ-15) as a standardized instrument for measuring QoL in these patients. Objective: This study aimed to assess QoL in patients with COMDs using an Indonesian version of the COMDQ-15. Methods: Seventy patients diagnosed with recurrent aphthous stomatitis (RAS), oral lichen planus, autoimmune blistering diseases (ABD), and cheilitis were included. Levels of QoL among different groups of disease were compared. Various potential factors influencing QoL were evaluated. Bivariate analysis was performed to identify factors associated with overall and specific aspects of QoL. Results: The mean total COMDQ-15 score was 20.83 ± 10.07. The highest scores were in the physical discomfort domain (8.76 ± 4.65), while the lowest was in the medication and treatment domain (2.13 ± 1.99). Physical discomfort was significantly associated with gender, major RAS, and cheilitis. Social and emotional aspects were significantly associated with age and ABD, while patient support was linked to employment status, RAS types, and cheilitis. Conclusions: The Indonesian version of the COMDQ-15 is a valid and reliable tool for assessing QoL in patients with COMDs.

## 1. Introduction

Chronic oral mucosal diseases (COMDs) encompass a range of long-standing lesions in the oral cavity that can result from infectious, inflammatory, and immunological disorders [1]. These conditions often have a significant impact on an individual’s quality of life (QoL) because of their chronic condition, clinical manifestations, recurrent episodes, prolonged treatment period, and the potential side effects of therapies. Immune-related chronic oral mucosal diseases, such as recurrent aphthous stomatitis (RAS), oral lichen planus (OLP), erythema multiform, systemic lupus erythematosus, and autoimmune blistering diseases (ABDs) such as pemphigus vulgaris, mucous membrane pemphigoid, are primarily caused by abnormal and dysregulated immune responses [2]. These conditions often present with varying degrees of oral inflammation, leading to functional disturbances such as difficulties in eating, speaking, and maintaining oral hygiene, which negatively affect the patient’s QoL and overall health, as well as the success of dental treatments [3,4,5]. In some conditions, there is a delay in diagnosis, misdiagnosis, and inappropriate treatment of oral mucosal diseases. This can occur because of several factors, including unrecognized oral mucosa diseases, whether primary oral diseases, those related to systemic disorders, or immune-mediated diseases [6,7]. This chronic oral mucosa disease can affect the patient’s QoL.

Quality of life, particularly in oral health, involves the interplay between oral conditions and aspects such as diet, nutrition, social interactions, emotional conditions, and psychological well-being. Discomfort, disability, and social or financial challenges arising from chronic oral conditions can severely disrupt these facets of life [8]. The Chronic Oral Mucosal Diseases Questionnaire-15 (COMDQ-15) is a validated and reliable instrument developed to measure QoL in patients with COMDs. This questionnaire, created using a patient-centered approach, has been extensively validated and proven effective in various settings [9,10].

Despite its widespread use and validation in different languages, the COMDQ-15 has not yet been adapted and validated for the Indonesian population. This study aims to translate the COMDQ-15 into Indonesian and ensure its cultural relevance and psychometric robustness for Indonesian patients with COMDs. By enabling patients to communicate objectively with their healthcare providers about their conditions, the Indonesian COMDQ-15 will help clinicians better understand the impact of chronic oral diseases on patients’ lives and improve treatment planning [2,11].

## 2. Methods

### 2.1. Participants

A total of 70 participants were enrolled in this study from the Oral Medicine Clinic at Universitas Indonesia Dental Hospital and Tangerang City Regional Public Hospital as a consecutive sample. The inclusion criteria were as follows: patients aged over 18 years with experience of COMDs such as recurrent aphthous stomatitis (RAS), oral lichen planus (OLP), autoimmune blistering diseases (ABD), and cheilitis simplex (CS), that was persistent and/or recurrent for at least 3 months, ability to understand and complete the questionnaire, and agreement to participate in this study. The diagnosis of the lesions was based on the patient’s history and clinical characteristics. Exclusion criteria included the following: persistent chronic neuropathic orofacial pain (e.g., post-traumatic trigeminal neuropathic pain, persistent idiopathic facial pain, or burning mouth syndrome), severe systemic illness (ASA 3 or more), and/or psychiatric conditions that may preclude research participation, such as schizophrenia. Participants were asked to complete the self-administered questionnaire after being examined by the dentist.

### 2.2. Study Design

This cross-sectional study was conducted from November 2022 to August 2023. The first stage involved testing the reliability and validity of the questionnaire, which had undergone a cross-cultural adaptation process and translation into Bahasa Indonesia. This study received ethical approval from the respective hospitals and the Dental Research Ethics Committee Faculty of Dentistry Universitas Indonesia (certificate number: 56/ethical approval/FKGUI/IX/2022 and 070/3819-Prclit/2022).

### 2.3. Cross-Cultural Adaptation, Reliability, and Validity of the Indonesian Version of COMDQ-15

The COMDQ-15 comprises 15 items addressing four major domains: pain and discomfort (PD; five items), medications and treatment (MT; three items), social and emotional (SE; five items), and patient support (PS; two items). Answers are graded on a 5-point Likert scale ranging from “not at all” (0) to “extremely” (4). The overall COMDQ score is obtained by summing the scores of all items, with a total range from 0 to 60, where higher scores indicate poorer QoL due to COMDs. The COMDQ-15 has undergone extensive validation studies, demonstrating its psychometric properties including content validity, convergent validity, discriminant validity, internal consistency, test–retest reliability, and responsiveness. It is suitable for use in both clinical and research settings to assess QoL in patients with COMDs.

The translation and cross-cultural adaptation process followed the internationally recommended six-step translation process. The first step included forward translation, which was conducted by one sworn translator and one expert in oral medicine, translating the questionnaire from English to Bahasa Indonesia. Panel experts from the field of oral medicine then reviewed the translated questionnaire. Following that, two different translators retranslated the questionnaire into English. A consensus meeting was held by a second panel of experts who reviewed and compared the back-translated version with the original version for clarity, cultural acceptability, and conceptual comparability. The final Indonesian version of the COMDQ-15 can be seen on Table 1. After that the questionnaire was tested in 30 patients to ensure reliability and validity.

To analyze the reliability of the Indonesian version of COMDQ-15, internal consistency reliability was determined by computing Cronbach’s alpha for each domain. Test–retest reliability was assessed by computing intraclass correlation coefficients (ICCs) using data from 30 patients who completed the COMDQ-15 after a 2-week interval. Cronbach’s alpha of 0.70 or above was considered statistically acceptable for group comparisons. ICC values can be classified into several categories based on their level of agreement, ranging from poor (<0.41) to excellent (>0.80). To analyze the validity of the Indonesian version of COMDQ-15, Spearman’s correlation was applied to examine the correlation between each item score and its total score. Items were considered valid if *p* < 0.05.

### 2.4. Statistical Analysis

The data distribution of domains and total scores of COMDQ-15 were assessed using the Kolmogorov–Smirnov test to determine the appropriate statistical tests for data analysis. The “PD” domain and the total COMDQ-15 scores showed a normal distribution, while the “MT”, “SE”, and “PS” domains were non-normally distributed.

Descriptive cross-sectional analyses were summarized using minimum score, maximum score, mean, and standard deviation. Categorical variables were represented using frequencies and percentages. Each domain and overall scores of COMDQ-15 were used as dependent variables. Bivariate analyses were performed to identify potential determinants of the total COMDQ-15 score and its domains using a parametric independent sample *t*-test for normally distributed variables (“PD” domain and total COMDQ-15 score) and a non-parametric Mann–Whitney U test for non-normally distributed variables (“MT”, “SE”, and “PS” domains), with a *p*-value of < 0.05.

## 3. Results

### 3.1. Validity and Reliability of COMDQ-15

The validity of the Indonesian version of the COMDQ-15 questionnaire was measured by finding a correlation between each item score and its total score using Spearman’s correlation, with a significant level (*p* < 0.05) as shown in Table 2.

To evaluate the reliability of the COMDQ-15, the questionnaire was tested and retested in 30 participants with a 2-week interval. The results indicated that each question of the Indonesian version of the COMDQ-15 was reliable, with a Cronbach’s alpha value of 0.848 and an ICC value of 0.705, indicating a good level of internal consistency for the Indonesian-adapted scale.

### 3.2. Participant’s Responses to the COMDQ-15

All 70 participants were included in the analyses since there were no missing data. The mean total COMDQ-15 score of the participants was 20.83 ± 10.07, with a maximum possible score of 60 (range: 2–47). Descriptive statistics for the total COMDQ-15 score and its domains are shown in Table 3.

The highest mean age was found in those suffering from OLP (56.6 years), followed by ABD (48.33 years) and herpetiform RAS, as shown in Table 4.

The distribution of gender based on the clinical classification of oral mucosal conditions is shown in Table 5. It was observed that a higher percentage of participants with each diagnosis were females, except for cheilitis, which had an equal percentage in both genders.

Bivariate analyses of patient characteristics and COMDQ-15 scores were conducted to determine the associated factors of QoL (Table 6 and Table 7). The mean age of all participants was 30.19 ± 13.95 years (range: 18–74 years), with most participants being under 35 years old (78.6%). Most participants were females (77.1%) and unemployed (81.4%).

The total COMDQ-15 score was significantly associated with gender (*p* = 0.004). The “PD” domain was significantly related to gender (*p* = 0.001), with female participants having higher mean total COMDQ-15 scores and “PD” domain scores, indicating a lower QoL outcome. The “MT” domain was not significantly associated with any demographic characteristics or clinical classifications. The “SE” domain was significantly related to age group (*p* = 0.032), with patients aged 35 years and older. The “PS” domain was significantly associated with employment status (*p* = 0.016). Unemployed participants had higher mean “PS” domain scores.

Bivariate analyses of disease type associated with domains and total COMDQ-15 score are shown in Table 7. Patients with RAS were divided into minor, major, and herpetiform RAS, with minor RAS being the most common clinical classification in this study group (64.3%), followed by cheilitis (25.7%) and oral lichen planus (7.1%). The total COMDQ-15 score was significantly associated with major RAS (*p* = 0.016) and cheilitis (*p* = 0.006).

The “PD” domain was significantly related to all types of RAS (0.019), specifically major RAS (*p* = 0.008), and cheilitis (*p* = 0.002). The “PS” domain was significantly related to all types of RAS (0.002), specifically minor RAS (0.000), herpetiform RAS (0.019), and cheilitis (0.045). Participants with minor RAS had higher mean “PS” domain scores, while those with herpetiform RAS and cheilitis had lower mean scores. The “SE” domain was significantly related to ABD (0.005). Those with ABD reported higher mean “SE” domain scores, indicating a worse QoL level. Conversely, participants with cheilitis had a lower mean total COMDQ-15 score and “PD” domain score. However, none of the disease groups had a significant association with the “MT” domain.

## 4. Discussion

Chronic oral mucosal diseases (COMDs) can have long-term effects not only on oral functions such as chewing, speaking, and aesthetics but also on overall body health, ultimately impacting a person’s QoL [12]. Chronic mucosal disease can cause pain and functional limitations that should be carefully diagnosed and treated to improve the patient’s QoL. Wasacz K. et al. state that oral mucosal diseases consist of various disorders, including RAS, OLP, and ABD, that can cause pain in the patient and negatively impact the patient’s QoL. Patients consider oral mucosa disease to be a serious disease, and it affects their lives in various ways [13]. Administering precise and proper questionnaires that provide a thorough image of the impact of oral disorders on patients can add valuable information to clinical practice [2]. The COMDQ-15 questionnaire, which underwent cross-cultural adaptation into the Indonesian language and psychometric analysis, can be used as an instrument to assess the QoL of individuals with COMDs.

This study revealed that the prevalence of COMDs was mostly observed in patients aged below 35 years, except for ABD and OLP. The average ages for each diagnosis (RAS, OLP, ABD, CS) in the present cohort were 27, 57, 48, and 25 years, respectively. These results align with the studies by Kridin and Schmidt (2021) and Li et al. (2023), which indicate that ABD can occur at any age, with most diagnoses between 45 and 65 years, and OLP typically affects those aged 40 years and older [14,15]. Al-Johani’s (2019) study also revealed that RAS was more common in patients aged 22.3 years and with an age range of 21–28 years old [16]. Additionally, our study found that the prevalence of COMDs is higher among female respondents, consistent with findings that OLP and RAS lesions are more common in women [17].

The mean total COMDQ-15 score for all participants was 20.83 (Table 3), with patients with major RAS showing the highest mean total score (34.33), followed by ABD 29.33 (Table 7). Ulcers in major RAS, and mucocutaneous blisters that quickly ulcerate in ABD, cause severe pain that significantly interferes with oral functions [18] and ultimately can interfere with social–emotional activities [7]. Previous studies have shown that ABD predominantly affects physical and emotional status, with facial involvement and lesion severity correlating well with lower QoL [19].

In our study, patients aged 35 years and older and those diagnosed with ABD showed significant impacts on their social and emotional (SE) conditions. Most COMDs have multifactorial etiologies, influenced by biological, social, economic, cultural, and environmental variables [20]. Other research states that the quality of life of patients with pemphigus vulgaris, an ABD, worsens in severe cases. Social support and emotional care are needed to improve the patient’s QoL [21]. Older individuals can acquire psychosocial determinants related to higher intention, social influence, self-efficacy, action planning, and coping planning, which are likely correlated with their oral problems [20].

Statistically significant results were obtained from the physical discomfort (PD) domain for major RAS (*p* < 0.05). In our research, there was a trend that major RAS had an impact on the SE domain, with the average COMDQ component score being the second highest after the PD domain. Previous studies revealed that RAS may develop symptoms such as pain, burning sensation, dysphagia, dysgeusia, and stinging pain [22]. These symptoms increase discomfort, especially in large and deep lesions such as RAS major, and also the number and size of the ulcer [23]. Additionally, our study found that respondents with RAS felt that the support from those around them significantly influenced their experience, as indicated by significant results in the patient support (PS) domain for minor and herpetiform RAS. This could be due to the recalcitrant nature of RAS, which cannot be completely cured; therefore, the goal of therapy is symptom alleviation, shortened recovery time, and prophylaxis against recurrence [24]. In this study, the second-highest mean PD score was for patients with OLP, one chronic oral mucosal disease. The results of another study showed that, besides PD, OLP had an impact on the MT and SE scores of the COMDQ [25].

We discovered that cheilitis significantly affected the patient’s QoL, particularly in the PD and PS domains. The characteristics of cheilitis simplex, described in previous studies, include cracked lips, fissures, or desquamation often caused by the habit of licking the lips. This habit leads to the loss of the natural protective layer, resulting in dryness and discomfort of the lips [26].

This study is constrained by the restricted time available for data collection and the challenges associated with securing a substantial number of participants. The small number of patients with COMDs and the time required for definitive diagnosis due to histopathological examination contributed to these challenges. Further research should involve collaboration with more oral medicine clinics to obtain a larger and more varied sample of respondents and types of COMDs.

## 5. Conclusions

The cross-cultural adaptation and translation of the COMDQ-15 questionnaire into Indonesian demonstrated its validity and reliability for assessing QoL in patients with COMDs. The study confirmed the effectiveness of the COMDQ-15 in dental clinic settings, highlighting its ability to provide a comprehensive overview of each domain of QoL (PD, MT, SE, and PS) in Indonesian patients with COMDs. Future research with larger and more diverse samples, extended data collection, and inclusion of various chronic oral lesions is recommended to validate these findings and improve patient care.

## Figures and Tables

**Table 1:** The original English version and the Indonesian version of COMDQ-15.

	English Version	Indonesian Version
Physical Discomfort		
PD1	How much do certain types of food/drink cause you discomfort (spicy food, acidic food)?	Seberapa jauh jenis makanan/minuman tertentu menyebabkan Anda tidak nyaman (makanan berbumbu/pedas, makanan asam)?
PD2	How much do certain food textures cause you discomfort (rough food, crusty food)?	Seberapa jauh tekstur makanan tertentu menyebabkan Anda tidak nyaman (makanan kasar, makanan keras)?
PD3	How much does the temperature of certain foods/drinks cause you discomfort?	Seberapa jauh suhu makanan/minuman tertentu menyebabkan Anda tidak nyaman?
PD4	How much does your oral condition lead to discomfort when carrying out your daily oral hygiene routine (brushing, flossing, mouthwash usage)?	Seberapa jauh kondisi mulut Anda menyebabkan rasa tidak nyaman saat melakukan rutinitas kebersihan rongga mulut sehari-hari (menyikat gigi, menggunakan benang gigi, menggunakan obat kumur)?
PD5	How much do you feel you need medication to help you with activities of daily life (talking, eating, etc.)?	Seberapa jauh Anda merasa membutuhkan obat-obatan untuk membantu Anda melakukan aktivitas sehari-hari (berbicara, makan, dll)?
Medication and Treatment		
MT1	How concerned are you about the possible side effects of the medications used to treat your oral condition?	Seberapa khawatirkah Anda tentang kemungkinan efek samping dari obat-obatan yang digunakan untuk merawat kondisi mulut Anda?
MT2	How much does it frustrate you that there is no single standard medication to be used in your oral condition?	Seberapa jauh membuat Anda frustrasi bahwa tidak terdapat satupun obat standar yang dapat digunakan pada kondisi mulut Anda?
MT3	How much does the use of the medication limit you in your everyday life (routine/the way you apply or take your medications)?	Seberapa jauh penggunaan obat-obatan membatasi Anda dalam kehidupan sehari-hari (rutinitas/cara Anda memakai/meminum obat)?
Social and Emotional		
SE1	How much does your oral condition get you down?	Seberapa jauh kondisi mulut Anda membuat Anda sedih?
SE2	How much does your oral condition cause you anxiety?	Seberapa jauh kondisi mulut Anda menyebabkan Anda cemas?
SE3	How much does the unpredictability of your oral condition bother you?	Seberapa jauh ketidakpastian kondisi mulut Anda mengganggu Anda?
SE4	How much does your oral condition make you pessimistic about the future?	Seberapa jauh kondisi mulut Anda membuat Anda pesimis tentang masa depan?
SE5	How much does your oral condition disrupt social activities in your life (social gatherings, eating out parties)?	Seberapa jauh kondisi mulut Anda mengganggu aktivitas sosial dalam kehidupan Anda (pergaulan sosial, pesta makan di luar)?
Patient Support		
PS1	How satisfied are you with the level of support and understanding shown to you by family regarding this oral condition?	Terkait kondisi mulut ini, seberapa puaskah Anda dengan tingkat dukungan dan pengertian yang ditunjukkan keluarga kepada Anda?
PS2	How satisfied are you with the level of support and understanding shown to you by friends/work colleagues regarding your oral condition?	Terkait kondisi mulut ini, seberapa puaskah Anda dengan tingkat dukungan dan pengertian yang ditunjukkan teman/rekan kerja kepada Anda?

**Table 2:** Correlation among the individual COMDQ-15 items and the total score.

	PD1	PD2	PD3	PD4	PD5	MT1	MT2	MT3	SE1	SE2	SE3	SE4	SE5	PS1	PS2	Total
PD1	-															
PD2	0.007 *	-														
PD3	0.040 *	0.027 *	-													
PD4	0.001 *	0.000 *	0.003 *	-												
PD5	0.017 *	0.120	0.024 *	0.007 *	-											
MT1	0.270	0.329	0.435	0.033 *	0.498	-										
MT2	0.086	0.264	0.058	0.076	0.359	0.008 *	-									
MT3	0.040 *	0.174	0.003 *	0.008 *	0.106	0.097	0.011 *	-								
SE1	0.086	0.026 *	0.001 *	0.003 *	0.080	0.149	0.074	0.001 *	-							
SE2	0.128	0.217	0.063	0.026 *	0.073	0.018 *	0.101	0.002 *	0.000 *	-						
SE3	0.084	0.181	0.079	0.021 *	0.033	0.032 *	0.036 *	0.001 *	0.000 *	0.000 *	-					
SE4	0.178	0.105	0.003 *	0.007 *	0.051	0.099	0.005 *	0.000 *	0.000 *	0.000 *	0.000 *	-				
SE5	0.032 *	0.036 *	0.003 *	0.004 *	0.006 *	0.466	0.049 *	0.001 *	0.000 *	0.000 *	0.000 *	0.000 *	-			
PS1	0.415	0.177	0.440	0.118	0.437	0.086	0.299	0.411	0.450	0.208	0.123	0.440	0.378	-		
PS2	0.474	0.397	0.340	0.373	0.443	0.377	0.265	0.337	0.446	0.182	0.160	0.373	0.350	0.000 *		
Total	0.000 *	0.000 *	0.000 *	0.000 *	0.000 *	0.108	0.011 *	0.000 *	0.000 *	0.000 *	0.000 *	0.000 *	0.000 *	0.298	0.154	-

* Correlation is significant at the 0.05 level (1-tailed).

**Table 3:** Descriptive statistics of the total COMDQ-15 sore and its domains (N = 70).

Variable		Min	Max	Max Possible Score	Mean	95% CI for Mean		SD	Median
						Lower	Upper		
Domains	PD: Physical discomfort	0	19	20	8.76	7.65	9.87	4.655	9.00
	MT: Medication and treatment	0	7	12	2.13	1.65	2.6	1.992	2.00
	SE: Social and emotional	0	20	20	6.17	4.88	7.46	5.424	5.00
	PS: Patient support	0	8	8	3.77	3.21	4.33	2.354	4.00
Total COMDQ-15 score		2	47	60	20.83	18.43	23.23	10.072	19.50

**Table 4:** Cross-tabulation of age (years) and clinical classification of immune-mediated oral mucosal conditions.

Clinical Classification	Age (Years)				
	Mean	95% CI for Mean		Std. Deviation	Median
		Lower	Upper		
Recurrent aphthous stomatitis	27.61	24.56	30.66	10.84	23.00
Major	23.00	20.52	25.48	1.00	23.00
Minor	27.44	24.07	30.82	11.22	23.00
Herpetiform	34.67	15.69	53.64	7.64	33.00
Oral lichen planus (OLP)	56.60	43.53	69.67	10.53	62.00
Autoimmune blistering diseases (ABD)	48.33	−9.20	105.86	23.16	42.00
Cheilitis	24.67	20.77	28.56	7.84	23.00

**Table 5:** Cross-tabulation of gender and clinical classification of immune-mediated oral mucosal conditions.

Clinical Classification	Male		Female		Total
	N	%	N	%	
Recurrent aphthous stomatitis	8	15.7%	43	84.3%	51
Major	0	0.0%	3	100.0%	3
Minor	8	17.8%	37	82.2%	45
Herpetiform	0	0.0%	3	100.0%	3
Oral lichen planus (OLP)	1	20.0%	4	80.0%	5
Autoimmune blistering diseases (ABD)	1	33.3%	2	66.7%	3
Cheilitis	9	50.0%	9	50.0%	18

**Table 6:** Bivariate analyses of demographic factors associated with domains and total COMDQ-15 scores (N = 70).

Demographic Factors	Categories	N (%)	PD		MT		SE		PS		Total Score	
			Mean (SD)	*p*	Mean (SD)	*p*	Mean (SD)	*p*	Mean (SD)	*p*	Mean (SD)	*p*
Gender	Male	16 (22.9)	5.44 (3.93)	^a^ 0.001 *	2 (1.67)	^b^ 0.989	4.31 (4.96)	^b^ 0.117	2.81 (2.26)	^b^ 0.062	14.56 (8.80)	^a^ 0.004 *
	Female	54 (77.1)	9.74 (4.42)		2.17 (2.09)		6.72 (5.48)		4.06 (2.33)		22.69 (9.74)	
Age Group	<35 years old	55 (78.6)	8.84 (4.77)	^a^ 0.787	2.02 (2.03)	^b^ 0.252	5.49 (5.21)	^b^ 0.032 *	3.98 (2.42)	^b^ 0.121	20.33 (10.30)	^a^ 0.429
	≥35 years old	15 (21.4)	8.47 (4.34)		2.53 (1.85)		8.67 (5.63)		3 (2)		22.67 (9.29)	
Employment	Employed	13 (18.6)	8.15 (4.86)	^a^ 0.608	1.72 (2.12)	^b^ 0.081	7.46 (5.98)	^b^ 0.347	2.38 (2.02)	^b^ 0.016 *	21 (11.31)	^a^ 0.946
	Unemployed	57 (81.4)	8.89 (4.64)		1.42 (1.93)		5.88 (5.30)		4.09 (2.32)		20.79 (9.88)	

^a^ *t*-test, ^b^ Mann–Whitney U test, * statistically significant (*p* < 0.05).

**Table 7:** Bivariate analyses of disease type associated with domains and total COMDQ-15 scores (N = 70).

Disease Groups	Categories	N (%)	PD		MT		SE		PS		Total Score	
			Mean (SD)	*p*	Mean (SD)	*p*	Mean (SD)	*p*	Mean (SD)	*p*	Mean (SD)	*p*
RAS: All types	Yes	51 (72.9)	9.55 (4.17)	^a^ 0.019 *	1.98 (1.99)	^b^ 0.253	5.94 (5.31)	^b^ 0.541	4.31 (2.27)	^b^ 0.002 *	21.78 (9.63)	^a^ 0.195
	No	19 (27.1)	6.63 (5.32)		2.53 (1.98)		6.79 (5.82)		2.32 (1.97)		18.26 (11.02)	
RAS: Major	Yes	3 (4.3)	15.67 (1.16)	^a^ 0.008 *	4.33 (3.79)	^b^ 0.260	11.67 (7.57)	^b^ 0.193	2.67 (3.79)	^b^ 0.525	34.33 (14.84)	^a^ 0.016 *
	No	67 (95.7)	8.45 (4.51)		2.03 (1.87)		5.93 (5.25)		3.82 (2.30)		20.22 (9.53)	
RAS: Minor	Yes	45 (64.3)	9.22 (4.06)	^a^ 0.310	1.91 (1.83)	^b^ 0.303	5.38 (4.84)	^b^ 0.160	4.67 (1.99)	^b^ 0.000 *	21.18 (8.85)	^a^ 0.725
	No	25 (35.7)	7.92 (5.56)		2.52 (2.24)		7.6 (6.20)		2.16 (2.12)		20.2 (12.14)	
RAS: Herpetiform	Yes	3 (4.3)	8.33 (3.06)	^a^ 0.873	0.67 (0.58)	^b^ 0.225	8.67 (8.15)	^b^ 0.562	0.67 (0.58)	^b^ 0.019 *	18.33 (10.70)	^a^ 0.664
	No	67 (95.7)	8.78 (4.73)		2.19 (2.01)		6.06 (5.33)		3.91 (2.31)		20.94 (10.11)	
OLP	Yes	5 (7.1)	10 (4.24)	^a^ 0.539	2.8 (2.59)	^b^ 0.543	9 (4.30)	^b^ 0.092	3.2 (1.30)	^b^ 0.505	25 (5.83)	^a^ 0.340
	No	65 (92.9)	8.66 (4.70)		2.08 (1.96)		5.95 (5.47)		3.82 (2.42)		20.51 (10.29)	
ABD	Yes	3 (4.3)	8.67 (4.04)	^a^ 0.973	3.67 (1.16)	^b^ 0.131	15.67 (4.51)	^b^ 0.005 *	1.33 (2.31)	^b^ 0.078	29.33 (6.51)	^a^ 0.136
	No	67 (95.7)	8.76 (4.71)		2.06 (1.99)		5.75 (5.09)		3.88 (2.31)		20.45 (10.07)	
Cheilitis	Yes	18 (25.7)	5.94 (5.64)	^a^ 0.002 *	2.5 (2.07)	^b^ 0.341	4.11 (4.23)	^b^ 0.069	2.78 (2.34)	^b^ 0.045 *	15.33 (12.06)	^a^ 0.006 *
	No	52 (74.3)	9.73 (3.87)		2 (1.97)		6.88 (5.64)		4.12 (2.28)		22.73 (8.63)	

^a^ *t*-test, ^b^ Mann–Whitney U test, * statistically significant (*p* < 0.05).

## Data Availability

The data presented in this study are available on request from the corresponding author.

## References

[1] Bukhari A.F., Farag A.M., Treister N.S. (2020). Chronic Oral Lesions. Dermatol. Clin..

[2] Rajan B., Ahmed J., Shenoy N., Denny C., Ongole R., Binnal A. (2014). Assessment of Quality of Life in Patients with Chronic Oral Mucosal Diseases: A Questionnaire-Based Study. Perm. J..

[3] Song J.S., Hyun H.K., Shin T.J., Kim Y.J. (2018). Effects of Dental Treatment and Systemic Disease on Oral Health-Related Quality of Life in Korean Pediatric Patients. BMC Oral Health.

[4] Baharvand M., Karami H., Mortazavi H. (2014). Evaluation of Life Quality in Patients with Oral Mucosal Lesions. Int. J. Exp. Dent. Sci..

[5] Al Ramil A.M., Lamfoon S., Mawardi H. (2023). Dental Implants for Patients with Oral Mucosal Diseases: A Narrative Review and Clinical Guidance. Dent. Med. Probl..

[6] Atkin P.A., Cowie R. (2024). Oral Mucosal Disease: Dilemmas and Challenges in General Dental Practice. Br. Dent. J..

[7] Nassab A.R.G., Navabi N., Pour M.M., Charrosta N., Hashemipour M.A. (2021). Quality of Life in Patients with Chronic Oral Mucosal Conditions: A Qualitative Research. Pesqui. Bras. Odontopediatria Clín. Integr..

[8] Villanueva-Vilchis M., Lopez-Rios P., Garcia I., Gaitan-Cepeda L. (2016). Impact of Oral Mucosa Lesions on the Quality of Life Related to Oral Health. An Etiopathogenic Study. Med. Oral Patol. Oral Cir. Bucal.

[9] Wiriyakijja P., Porter S., Fedele S., Hodgson T., McMillan R., Shephard M., Ni Riordain R. (2020). Development and Validation of a Short Version of Chronic Oral Mucosal Disease Questionnaire (COMDQ-15). J. Oral Pathol. Med..

[10] Ni Riordain R., Mccreary C. (2012). Further Reliability and Responsiveness of the Chronic Oral Mucosal Diseases Questionnaire: Quality of Life Questionnaire Testing. Oral Dis..

[11] Wiriyakijja P., Porter S., Fedele S., Hodgson T., McMillan R., Shephard M., Ni Riordain R. (2021). Health-Related Quality of Life and Its Associated Predictors in Patients with Oral Lichen Planus: A Cross-Sectional Study. Int. Dent. J..

[12] Shirzad A., Bijani A., Mehryari M., Motallebnejad M., Mohsenitavakoli S. (2018). Validity and Reliability of the Persian Version of the Chronic Oral Mucosal Diseases Questionnaire. Casp. J. Intern. Med..

[13] Wasacz K., Chomyszyn-Gajewska M., Hukowsha D. (2022). Oral Heatlh-Related Quality of Life (Ohrqol) in Polish Adults with Periodontal Diseases, Oral Mucosal Diseases and Dental Caries. Dent. Med. Probl..

[14] Kridin K., Schmidt E. (2021). Epidemiology of Pemphigus. JID Innov..

[15] Li C., Tang X., Zheng X., Ge S., Wen H., Lin X., Chen Z., Lu L. (2020). Global Prevalence and Incidence Estimates of Oral Lichen Planus. JAMA Dermatol..

[16] Al-Johani K. (2019). Prevalence of Recurrent Aphthous Stomatitis among Dental Students: A Cross Sectional Study. J. Contemp. Dent. Pract..

[17] Radwan-Oczko M., Sokół I., Babuśka K., Owczarek-Drabińska J.E. (2022). Prevalence and Characteristic of Oral Mucosa Lesions. Symmetry.

[18] Alshami M.L., Aswad F., Abdullah B. (2022). A Clinical and Demographic Analysis of Oral Pemphigus Vulgaris: A Retrospective Cross-Sectional Study from 2001 To 2021. Health Sci. Rep..

[19] Sebaratnam D.F., Mcmillan J.R., Werth V.P., Murrell D.F. (2012). Quality of Life in Patients with Bullous Dermatoses. Clin. Dermatol..

[20] De Abreu M.H.N.G., Cruz A.J.S., Borges-Oliveira A.C., Martins R.D.C., Mattos F.D.F. (2021). Perspectives on Social and Environmental Determinants of Oral Health. Int. J. Environ. Res. Public Health.

[21] Salehi M.R., Tabesh A., Mahdian A.M. (2023). Does Pemphigus Vulgaris Affect Oral Health Related Quality of Life?. Nat. Life Sci. Commun..

[22] Siml Q., Mvad S., Amcd M., Ptd O., Bcdv G., Éjdd S. (2018). Recurrent Aphthous Ulceration: An Epidemiological Study of Etiological Factors, Treatment and Differential Diagnosis. An. Bras. Dermatol..

[23] Rivera C., Pasten M.M., Munoz E.N., Olivos R.H. (2022). Recurrent Aphthous Stomatitis Affects Quality of Life. A Case-Control Study. Clin. Cosm. Investig. Dent..

[24] Manfredini M., Guida S., Giovani M., Lippolis N., Spinas E., Farnetani F., Dattola A., Di Matteo E., Pellacani G., Giannetti L. (2021). Recurrent Aphthous Stomatitis: Treatment and Management. Dermatol. Pract. Concept..

[25] Saberi Z., Tabesh A., Darvish S. (2022). Oral Health-Related Quality of Life in Erosive/Ulcerative Oral Lichen Planus Patients. Dent. Res. J..

[26] Lugović-Mihić L., Pilipović K., Crnarić I., Šitum M., Duvančić T. (2018). Differential Diagnosis of Cheilitis—How to Classify Cheilitis?. Acta Clin. Croat..

